# Heat exhaustion, domestic heat exposure and socioeconomic disparities among older adults: a cross-sectional mediation analysis from Germany

**DOI:** 10.1186/s12939-026-02934-8

**Published:** 2026-07-07

**Authors:** Catherina Heinken, Saskia Muellmann, Tilman Brand, Thomas Behrens, Swaantje Casjens, Jacob Spallek, Jonas Frost, Nadine Glaser, Nils Opel, Janka Massag, Rafael Mikolajczyk, Michael Gekle, Irene Moor, Oliver Tüscher, Michael Heuser, Benjamin Schüz, Hajo Zeeb

**Affiliations:** 1 University of Bremen, Health Sciences, Bremen, Germany; 2 Leibniz ScienceCampus Digital Public Health (LSC DiPH), Bremen, Germany; 3 Leibniz Institute for Prevention Research and Epidemiology – BIPS, Achterstraße 30, 28359 Bremen, Germany; 4 Medical Faculty, Institute for Prevention and Occupational Medicine of the German Social Accident Insurance of the Ruhr University Bochum (IPA), Ruhr University Bochum, Bochum, Germany; 5 Department of Public Health, Brandenburg University of Technology Cottbus-Senftenberg, Senftenberg, Germany; 6 Institute for Medical Epidemiology, Biometrics, and Informatics, Interdisciplinary Centre for Health Sciences, Medical Faculty of the Martin, Luther University Halle- Wittenberg, Magdeburger Str. 8, 06112 Halle (Saale), Germany; 7 Department of Psychiatry & Neuroscience, Charité Universitätsmedizin Berlin, Berlin, Germany; 8 Julius Bernstein Institute of Physiology, Martin LutherUniversität Halle-Wittenberg, 06112 Halle (Saale), Germany; 9 Institute of Medical Sociology, Interdisciplinary Centre for Health Sciences, Medical Faculty of the Martin, Luther University Halle-Wittenberg, Magdeburger Str. 8, 06112 Halle (Saale), Germany; 10 German Center for Mental Health (DZPG), Site Halle-Jena-Magdeburg, Halle (Saale), Germany; 11 Leibniz Institute for Resilience Research Mainz gGmbH, Mainz, Germany; 12 Department of Internal Medicine IV, University Hospital Halle (Saale), Martin-Luther-University Halle-Wittenberg, Halle, Germany

**Keywords:** Indoor temperature, Heat, Elderly, Social inequalities, Climate change, Domestic

## Abstract

**Background:**

Older adults are particularly vulnerable to the effects of heatwaves and extreme heat due to physiological changes often exacerbated by chronic diseases and decreased mobility. Although cool indoor temperatures can moderate the health consequences of extreme outdoor heat to some extent, it is important to recognize that access to cool indoor temperatures is distributed unequally across population groups. This study aimed to assess the mediating effect of (indoor) domestic heat exposure on the relationship between socioeconomic position and heat exhaustion in older adults.

**Methods:**

In August 2024, 10,000 participants from the DigiHero cohort were invited to take part in an online survey. Of the 5,026 respondents, persons aged 65 or older were included in the analysis (*n* = 1,457). Domestic heat exposure was assessed through self-reported indoor temperatures during the day and at night, as well as the availability of cooler rooms at night. Heat exhaustion was measured using self-reported symptoms. Income and education were used as indicators for socioeconomic position. We applied structural equation modelling to assess the total impact of socioeconomic position (education/income) on heat exhaustion and the portion of this effect mediated through domestic heat exposure. Additionally, the analyses were stratified by sex/gender and degree of urbanization.

**Results:**

In the study sample, 54% were female; mean age was 72.8 (SD = 5.1); 50% had a high education level; and 70% were not exposed to domestic heat. Lower income and education were linked to more frequent symptoms of heat exhaustion. Lower income, but not education, was associated with higher domestic heat exposure which mediated the income–heat exhaustion relationship (total effect β = -0.09 (-0.14, -0.03); indirect effect β = -0.01 (-0.02, -0.003)). Sex/gender-stratified analyses showed these associations only among women. Sensitivity analyses indicated a stronger mediation in urban settings.

**Conclusion:**

The analysis revealed that lower income result in more frequent symptoms of heat exhaustion in older women, partially due to differential domestic heat exposure. Potential sex/gender differences in the effects of heat exposure warrant further exploration.

**Supplementary Information:**

The online version contains supplementary material available at 10.1186/s12939-026-02934-8.

## Background

Anthropogenic climate change is one of the greatest challenges to human health in the current century [1]. Projections indicate an increase in the frequency of heat waves and extreme heat [2], which has already resulted in approximately 11,000 heat-related deaths in the record-breaking summer of 2018 and 9,100 deaths in Germany in 2022 [3]. Apart from mortality, heat-related health outcomes span a continuum, including symptoms like heat edema, heat cramps, heat exhaustion, and heat stroke [4]. Heat exhaustion is a condition characterized by elevated body temperature impaired with profuse sweating, changes in the mental status, dizziness, nausea, and headaches [5]. It is a condition that might indicate very early symptoms of heat stroke [4, 5].

Older adults (aged 65 or older) are more vulnerable to heat due to an interplay of physiological changes and their general health status, which is often impaired with one or more chronic conditions – most importantly cardiovascular conditions [6, 7]. It has been demonstrated that the administration of (multiple) commonly prescribed medications can impede the body’s capacity to adapt to elevated temperatures [6]. This phenomenon is particularly salient among older adults who are afflicted with multiple morbidities. Consequently, the elderly population bears the greatest proportion of heat-related mortality, with the rate (per million) in 2023 being 13, 127, and 1,102 for the age groups 0–64, 65–79, and 80+, respectively [8]. The heat-related mortality of older adults (65+) increased by 85% worldwide when comparing the years 2000–2004 to 2017–2021 [2]. In a study utilizing objective heat measures of outdoor temperature in the US, older adults (65+) who identified as People of Color, did not have a health insurance, or were living below the poverty line, experienced a higher mean outdoor temperature in 2015–2019 compared to people identifying as non-Hispanic White, insured individuals, and those not living below the poverty line [9].

Prior research has shown a higher heat-related health burden among women as compared to men. For example, in the extremely hot summer of 2003 the mortality rates in women were higher than in men in Spain and France [10]. This finding was corroborated by numerous subsequent studies which also reported increased heat-related mortality among women (e.g., [11, 12]). However, it should be noted that this finding was not uniform across all studies [10, 13]. Recently, for the summer of 2022, Ballester, Quijal-Zamorano [14] estimated a 56% higher heat-related crude mortality rate among women in Europe and a 37% higher heat-related crude mortality rate among women in Germany compared to men. Additionally, Slesinski, Matthies-Wiesler [15] found women to be more likely to report subjective heat stress than men. Nevertheless, the biological and social causes of disparities between women and men in heat-related health outcomes are not yet fully understood.

Existing evidence indicates that elevated outdoor temperatures are associated with increased mortality [3, 16], setting population groups at risk, which are exposed to heat, e.g., those living in densely populated urban areas or those working outside [6]. An increase in outdoor temperature of 1 °C has been found to be associated with an increase in indoor temperatures of 0.22 °C in London [17] and 0.36 °C in Montreal [7], with considerable variation between building characteristics [7], suggesting that housing conditions play an important role in mitigating outdoor heat. Besides outdoor temperatures, studies have reported that elevated indoor temperatures were associated with adverse heat-related health outcomes [18]. Since individuals aged 65 and older spend more time at home than younger age groups [19], their exposure to indoor temperatures is correspondingly higher.

Although southern Europe currently has the highest heat-related mortality rates compared with the rest of Europe [8, 14, 20], annual excess heat-related mortality in Germany is projected to increase by at least 2.5 times — without accounting for demographic changes such as expected population ageing — based on a model that assumes global warming of 2.7 °C by 2100 and current CO₂ emission trends continuing until 2050 [21]. Previous research has indicated variations of heat-related health risks within countries according to the degree of urbanization. A study from Spain showed has shown that heat waves led to higher mortality in urban areas as compared to rural areas [22]. Likewise, results from Germany revealed higher heat-related mortality risks in districts with greater urbanization and less green space [23].

Regarding social disparities in heat exposure, Osberghaus and Abeling [24] reported that low-income households in Germany are less well protected against heat waves, and further explained that adapting to heat technically, e.g., by air conditioning, would imply substantial costs for the household. Furthermore, another study from Germany showed that low-income house owners, and particularly retirees, live more frequently in older buildings that are presumably less well insulated [25].

Overall, comparatively little research has examined the role of indoor/domestic temperatures during heat waves and their health consequences in the European context, and more specifically, how social disparities in domestic heat exposure may contribute to health inequality. Hence, the aim of this study was to assess the association between income/education and heat exhaustion among older adults (65+) as well as the mediating effect of domestic heat exposure, addressing the following research question: To what extent does domestic heat exposure mediate the effect of socioeconomic position on heat exhaustion in older German adults (65+)? Given the well-documented relationship between socioeconomic deprivation and poor health, including cardiovascular diseases [26], we hypothesized that income and education (as indicators for socioeconomic position) affect heat exhaustion and that domestic heat exposure mediates these associations. To ensure the consistency of the associations, we stratified the analysis by sex/gender and degree of urbanization.

## Methods

### Study design and data

Data were collected in the population-based German digital cohort study DigiHero (DigiHero, DRKS registration ID: DRKS00025600). Initiated in 2021 in the federal state of Saxony-Anhalt, DigiHero was subsequently extended to all German federal states through multiple recruitment waves. Participants born between 1936 and 2003 (aged 18 to 85 in 2021) were recruited by post. Postal addresses were obtained from local registration offices, local service providers or a federal data portal, in accordance with German data protection rules. Study registration took place online via the study website. After registering, participants were invited by email to complete a baseline assessment including sociodemographic variables such as age, sex/gender, education, occupation and household income. All registered participants (*n* = 128,608 as of 2 April 2025) are subsequently contacted regularly to take part in online surveys on various health topics. Ethical approval was granted by the Ethics Committee of the Martin Luther University Halle-Wittenberg (2020-076). All participants received written information about the study and gave informed consent. A detailed description of the DigiHero cohort study has been published elsewhere [27]. For this analysis, data were obtained from the baseline assessment and a survey on heat protection and health conducted in August 2024. To this end, a subsample of 10,000 DigiHero participants, stratified by federal state, sex/gender and age, was randomly selected to participate in the survey. Of those invited, 5,026 participants completed the survey, resulting in a response of 50%. To maintain consistency of income as an indicator of socioeconomic position within the sample [28], we analyzed data from participants aged 65 years and above and who were no longer employed.

### Measures

#### Heat exhaustion

Adverse health outcomes through extreme heat events were defined as the sum of symptoms of heat exhaustion experienced during the recent summer period. Included symptoms were dizziness, headache, and shortness of breath. These symptoms were self-reported and were considered eligible if the participant would trace their symptoms back to heat exposure. All three symptoms of heat exhaustion were ranked on a scale from ‘never’ (0) to ‘very often’ [4]. Responses relating to the three symptoms were summed up to give a score ranging from 0 to 12, with a higher score indicating a greater burden of adverse health effects from heat. The items used demonstrated an internal consistency of Cronbach’s alpha = 0.60. The English version of the questionnaire items used is available in Table A1 (additional files).

#### Domestic heat exposure

Domestic heat exposure was defined as self-reported exposure to high night-time temperatures, limited access to cool environments at night and uncomfortable indoor temperatures during the day. Elevated night-time temperatures were defined as temperatures in a participant’s sleeping room exceeding 24 degrees Celsius after a very warm day. Limited access to cooler environments applied to those who reported an elevated temperature in their bedroom and did not report having access to cooler areas in their home. Uncomfortable indoor temperatures during the day were identified based on respondents negating that the areas or rooms in which they usually stayed at home were comfortable on very warm days. All indoor heat exposure items were self-reported. An exposure score (0–3) was calculated by summing the presence (1 = yes) of elevated night-time temperature, limited access to cool environments at night, and uncomfortable indoor temperatures during the day.

#### Socioeconomic indicators

Equivalized net income was determined by the weighted ratio of the average income within the income groups collected and the number of household members. All household members other than the study participant were assigned a weight of 0.5 if aged over 14 and 0.3 if aged 14 or under [29]. Educational status was classified using the International Standard Classification of Education (ISCED-2011) [30]. The aim was to classify educational levels into three groups, thereby assigning approximately one third of participants to each group. Therefore, we grouped ISCED classes 1–4 (low education), 5–6 (medium education) and 7–8 (high education). Given that 50% of participants hold a master’s degree or higher, it was not possible to form exact tertiles. Age was defined based on participants’ birth year.

#### Stratification variables

Sex/Gender was assessed by asking the survey participants to specify whether they were: male, female, or diverse. We use the term sex/gender throughout the paper because the survey question did not clearly distinguish between participants’ biological sex and their gender identity. A second reason was that both biological and social factors may affect exposure to heat and reporting of heat exhaustion. Degree of urbanization was defined using the classification of the Federal Institute for Urban and Regional Research [31]. Participants were assigned to administrative districts based on their postal codes and districts were classified as urban (> 150 inhabitants per km²) or rural (≤ 150 inhabitants per km²).

### Analysis

**Fig. 1:**
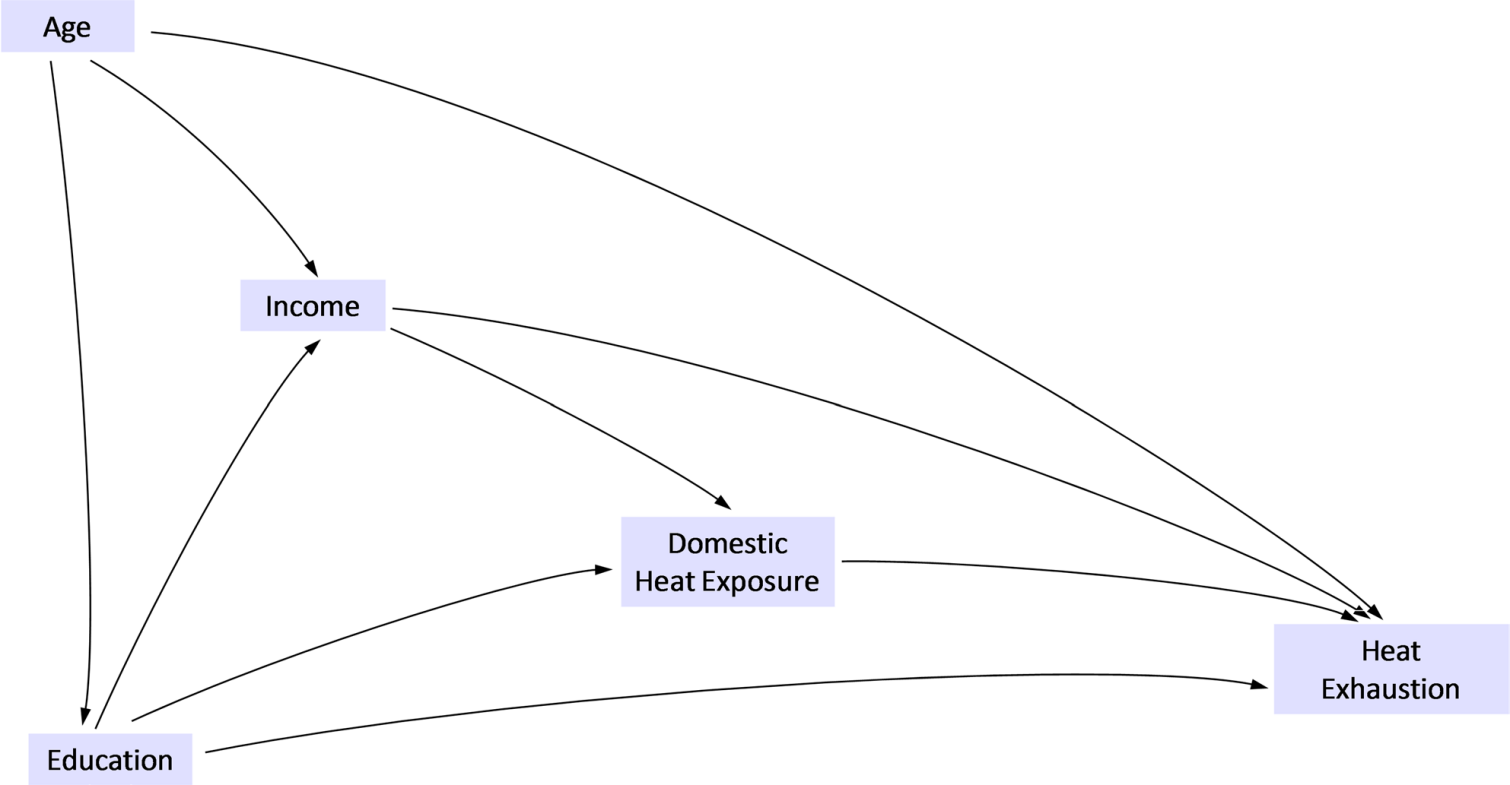
Hypothesized structural equation model

First, we specified a linear structural equation model (SEM) to reflect our assumptions about the relationships between socioeconomic position, exposure to domestic heat, and heat exhaustion among older adults in Germany (see Fig. 1). The same model was used for the stratified analysis by sex/gender and degree of urbanization. The second step involved using path analysis to investigate the mediation effects of domestic heat exposure on the association between income/education and heat exhaustion. The model can be described as follows [32]:

1 $$\:a*b\:+\:{c}^{{\prime\:}}=\:c$$

Where a*b is the indirect effect (the product of the coefficients), c’ is the direct effect, and c is the total effect of exposure X (income, education) on outcome Y (heat exhaustion) [33]. The traditional approach to determining the fit of SEM models is to use the chi-square value; however, this value is sensitive to small sample sizes and may not differentiate sufficiently between models that fit well and those that do not when analyzing small sample sizes [34]. According to Hooper, Couglan [34] we decided to utilize the root mean square error of approximation (RMSEA) as an absolute fit index and the comparative fit index (CFI) as an incremental fit index [34]. The value of the RMSEA can be considered to indicate a good fit if it is smaller than 0.06. The CFI describes a good fitting model when its value is ≥ 0.95 [34].

Within SEM, the term ‘effect’ is used solely in its technical sense, without implying a causal relationship.

The estimates are reported as standardized betas (β) with 95% confidence intervals (CI) to ensure comparability of the effects within the models; confidence intervals were bootstrapped with 1,000 replications. Ordinal variables were treated as continuous, as simulation studies have indicated that, in medium to large samples (*n* > 500), parameters estimated in robust maximum-likelihood are largely comparable to those from categorical estimators [35]. Missing data in any of the included variables (in approximately 15% of all cases) were handled using full information maximum likelihood [36]. Since this method assumes missing data are missing at random (MAR) [37], we performed an additional sensitivity analysis using only complete cases. All statistical analyses were conducted using Stata (Release 18, College Station, TX: StataCorp LLC), and specifically the SEM command, which uses a maximum likelihood function for estimation [37].

## Results

### Participant characteristics

Of the 5,026 respondents, 1,457 met the inclusion criteria. Of these, 54% were women and 46% were men (Table 1). The mean age was 72.8 (SD = 5.1), with the majority (50%) falling into the high (ISCED 7–8) educational status group. Within the overall sample, and among both men and women, more than half of the participants reported an equivalized net household income of €2,000 or more per month. Around 70% were not exposed to domestic heat during hot summer days. The mean score for heat exhaustion experienced during the recent summer heat was 1.2 (SD = 1.9) for men, 1.9 (SD = 2.4) for women and 1.6 (SD = 2.2) for the total sample. Approximately half of our sample lived in urban areas.

**Table 1:** Sample characteristics of eligible DigiHero participants

	Men	Women	Total
N	668 (45.8%)	789 (54.2%)	1457 (100.0%)
**Age**			
Mean (SD)	73.2 (5.3)	72.4 (4.8)	72.8 (5.1)
**Education according to ISCED**			
Low (ISCED 1–4)	139 (22.0%)	269 (36.2%)	408 (29.7%)
Medium (ISCED 5–6)	136 (21.5%)	140 (18.8%)	276 (20.1%)
High (ISCED 7–8)	357 (56.5%)	335 (45.0%)	692 (50.3%)
**Equivalized net household income [€]**			
Less than 2,000	219 (35.4%)	310 (43.1%)	529 (39.5%)
2,000–3,000	196 (31.7%)	226 (31.4%)	422 (31.5%)
3,000–4,000	122 (19.7%)	115 (16.0%)	237 (17.7%)
4,000 and more	82 (13.2%)	69 (9.6%)	151 (11.3%)
**Domestic Heat Exposure** ^1^			
0	470 (70.5%)	529 (67.1%)	999 (68.7%)
1	170 (25.5%)	206 (26.1%)	376 (25.8%)
2–3	27 (4.0%)	53 (6.7%)	80 (5.5%)
**Frequency of Symptoms of Heat Exhaustion** ^**2**^			
Mean (SD)	1.2 (1.9)	1.9 (2.4)	1.6 (2.2)
**Type of Residency**			
Urban^3^	328 (49.2%)	356 (45.1%)	684 (47.0%)
Rural^4^	338 (50.8%)	433 (54.9%)	771 (53.0%)

^1^ Sum of exposures to elevated temperatures during the night, limited access to cooler rooms during the night, and presence of uncomfortable indoor temperatures at home during the day

^2^ Included symptoms were dizziness, headache, and shortness of breath

^3^ > 150 inhabitants per km^2^

^4^ ≤ 150 inhabitants per km^2^

SD = Standard Deviation; ISCED = International Standard Classification of Education

### SEM model

**Fig. 2:**
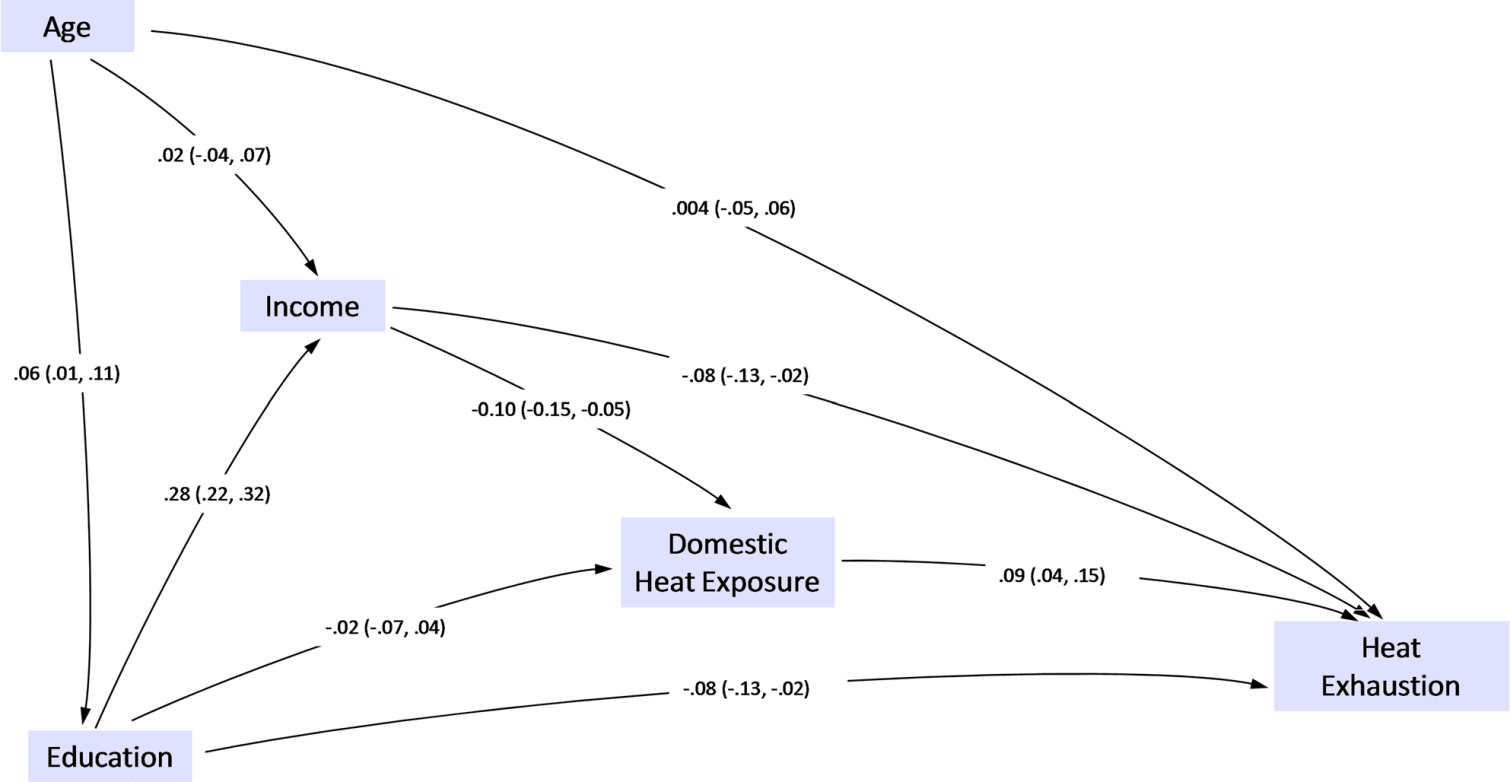
Standardized direct effects (β; 95% CI) in the main hypothesized structural equation model (*n* = 1,457)

**Fig. 3:**
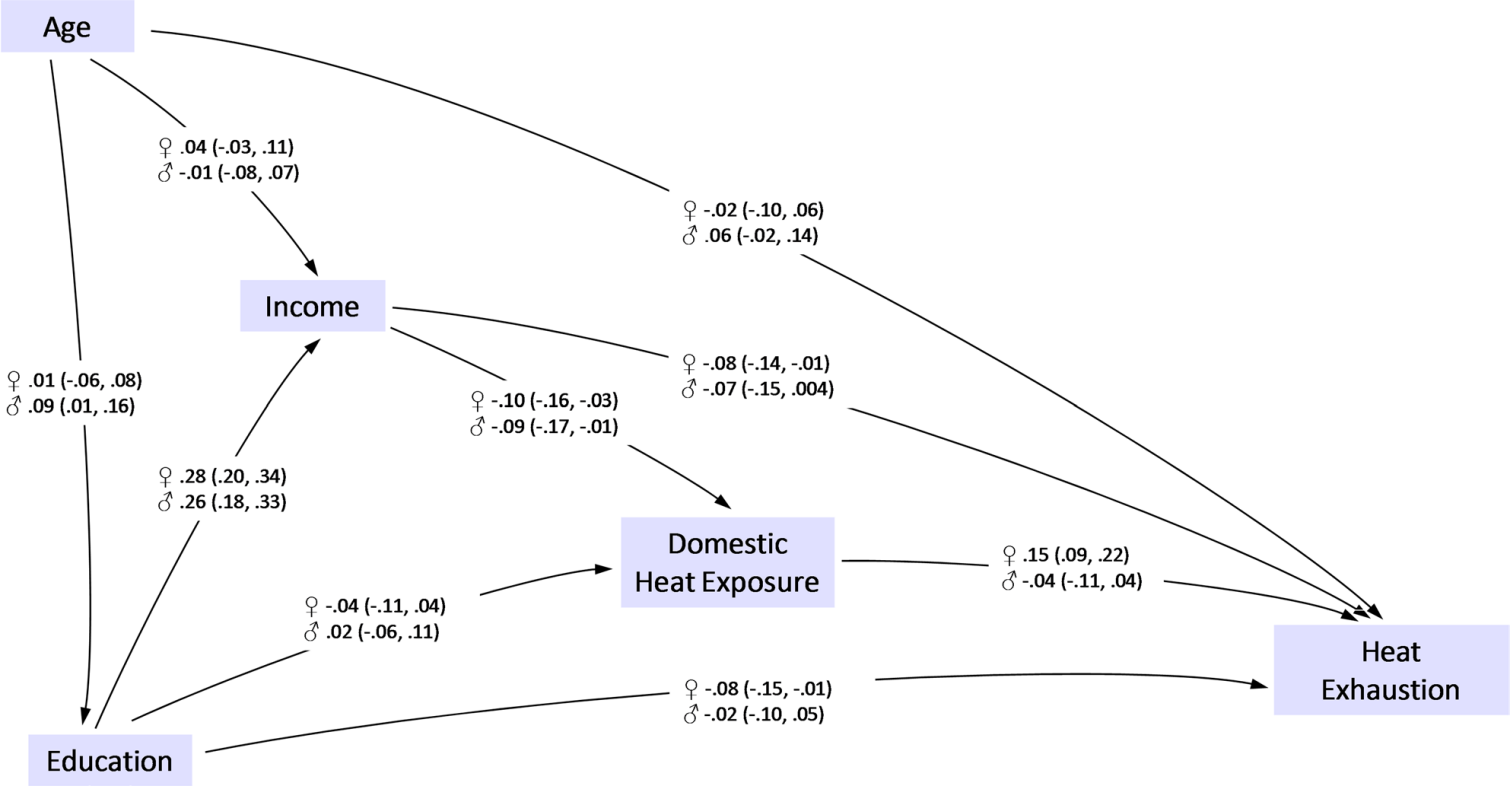
Standardized direct effects (95% CI) in the sex/gender-stratified hypothesized structural equation model (women: *n* = 789, men: *n* = 668)

Figures 2 and 3 show the results of structural equation modeling. The main model revealed an RMSEA of 0.00 and a CFI of 1.00, indicating an excellent fit for our SEM model. A nested model comparison was conducted to assess potential sex/gender-related differences in structural relationships. Therefore, we tested whether constraining the structural path between domestic heat and heat exhaustion to equality for men and women significantly reduced model fit. The likelihood-ratio test indicated a significant decline in fit (χ²(19) = 131.42, p = < 0.01), hence this structural path differed between men and women. Consequently, we estimated stratified models. The models stratified by sex/gender both revealed an RMSEA of 0.00 and a CFI of 1.00, which again indicates an excellent model fit.

In the main model (Fig. 2), exposure to domestic heat was positively associated with heat exhaustion (β = 0.09; 95% CI: 0.04, 0.15). Income was negatively associated with both heat exposure (β = -0.10; 95% CI: -0.15, -0.05) and heat exhaustion (β = -0.08; 95% CI: -0.13, -0.02), indicating lower exposure to domestic heat and a lower frequency of heat exhaustion in households with higher income. Education was positively associated with household income (β = 0.28; 95% CI: 0.22, 0.32) and showed a negative direct effect on adverse heat exhaustion (β = −0.08; 95% CI: −0.13, − 0.02). This suggests that heat exhaustion occurred less frequently among higher-educated participants. However, there was no direct effect of education on domestic heat exposure (β = -0.02; 95% CI: -0.07, 0.04). Age was not associated with heat exhaustion nor with income, but with education (β = 0.06; 95% CI: 0.01, 0.11).

### Paths analysis

The path analysis showed both a total effect of income on heat exhaustion (β = -0.09 (95% CI: -0.14, -0.03); Table 2), and a negative direct effect on domestic heat exposure (Fig. 2). Education had a total effect on heat exhaustion (β = -0.10; 95% CI: -0.16, -0.05), but no direct effect on domestic heat exposure, as described above. There was an indirect effect of income on heat exhaustion via domestic heat exposure (β = -0.001; 95% CI: -0.01, -0.003). The ratio of the indirect to total effect (RIT) was 0.10, indicating that 10.0% of the association between income and heat exhaustion was mediated by exposure to domestic heat. Education had no indirect effect on heat exhaustion via domestic heat exposure (β = -0.001; 95% CI: -0.01, 0.003).

### Sex/gender-stratified models

Figure 3 shows the results of the sex/gender-stratified structural equation modeling. Among women, domestic heat exposure had a positive direct effect on heat exhaustion (β = 0.15; 95% CI: 0.09, 0.22), whereas among men, this was not the case (β = -0.04; 95% CI: -0.11, 0.04). Income was associated with lower domestic heat exposure in both women (β = -0.10; 95% CI: -0.16, -0.03) and men (β = -0.09; 95% CI: -0.17, -0.01). Income had a negative direct effect on heat exhaustion in women (β = -0.08; 95% CI: -0.14, -0.01). Education showed no effects on domestic heat exposure in both women (β = -0.04; 95% CI: -0.11, 0.04) and men (β = 0.02; 95% CI: -0.06, 0.11). Age was not associated with heat exhaustion among men and women. However, the effect of age on heat exhaustion was more pronounced in men, and the effects differ in direction between men and women. Age was not associated with income in both models but with education in men (β = 0.09; 95% CI: 0.01, 0.16). The estimates of the total and indirect effects of income and education on heat exhaustion showed a stronger association among women compared to men in the path analysis (Table 2). Specifically, the small direct effect of domestic heat exposure on heat exhaustion within men suggested that there was no mediation via this exposure.

**Table 2:** Standardized total and indirect effects from path analysis

Path	All Participants (*n* = 1,457)	Men (*n* = 668)	Women (*n* = 789)
*Total effect on heat exhaustion*	β *(95% CI)*	β *(95% CI)*	β *(95% CI)*
Income	-0.09 (-0.14, -0.03)	-0.07 (-0.14, 0.01)	-0.09 (-0.16, -0.02)
Education	-0.10 (-0.16, -0.05)	-0.04 (-0.12, 0.03)	-0.11 (-0.18, -0.03)
*Indirect effect on heat exhaustion (via domestic heat exposure)*	β *(95% CI)*	β *(95% CI)*	β *(95% CI)*
Income	-0.01 (-0.02, -0.003)	0.003 (-0.003, 0.02)	-0.02 (-0.03, -0.004)
Education	-0.001 (-0.01, 0.003)	-0.001 (-0.01, 0.002)	-0.01 (-0.02, 0.01)

### Stratification by degree of urbanization

A total of 1,455 persons reported information on their residency. Of these, 47.0% lived in rural areas; among rural residents 48.0% were men compared with 43.8% among urban residents. While urban residents reported greater exposure to domestic heat, the frequency of heat exhaustion did not differ between urban and rural residents (Table A2, additional files). The SEM and path analysis revealed consistent patterns across both settings, although the effect from domestic heat exposure on heat exhaustion was slightly stronger among urban residents (β = 0.12; 95% CI: 0.05, 0.19 vs. β = 0.04; 95% CI: -0.03, 0.12). In addition, with a RIT = 0.10 in urban settings compared to RIT = 0.05 in rural settings, the results suggest that a larger share of income-related disparities in heat exhaustion could be attributed to domestic heat exposure in urban settings (Table A3, additional files).

### Complete case analysis

Evaluation of the main and sex/gender-stratified models using only complete cases showed results very similar to those obtained with full information maximum likelihood estimation (Table A4, additional files), supporting the assumption that missing data were missing (completely) at random.

## Discussion

This study examined the relationship between socioeconomic position and heat exhaustion among older adults, and more specifically the mediating effect of domestic heat exposure. Our analysis revealed that higher income was associated both directly and indirectly with lower frequencies of heat exhaustion. Exposure to domestic heat mediated about 10% of this association. However, the results of the sex/gender-stratified models suggested that the findings were observable only in women. In addition, a comparison by degree of urbanization revealed that domestic heat exposure contributed more to income disparities in heat exhaustion in urban areas as compared to rural areas.

During the surveyed summer period in 2024, heat-related mortality rates in Germany were moderately high (2,800; 95% prediction interval: 1,000, 5,000) compared to the years 1992 to 2023. The average temperature was 2.2 °C higher than the international reference period for Germany, and an average of 6.8 heat weeks were observed, with substantial variation between regions [38]. Here, a heat week is defined as an average weekly temperature of 20 °C or higher, the point at which the exposure-response relationship (weekly average temperature vs. relative mortality rate) rises steeply across all ages [39]. Literature on heat-related morbidity remains scarce; however, one report found an increase in the number of persons reporting to have experienced heat-related health problems when comparing 2023 to 2024 [40]. Although an der Heiden [38] evaluated the entire summer half-year and our definition of “summer” was less precise, these findings suggest a relatively high burden of heat-related health outcomes in 2024, particularly among older adults. Nevertheless, the observed numbers are comparably moderate in comparison to the extremely hot summers in 2003 and 2018 [38, 40].

Our analysis revealed an association between domestic heat exposure and heat exhaustion among women, whereas this association was found to be smaller and not statistically significant among men. The findings of several studies imply that women are at greater risk of adverse health effects when experiencing heat stress. Navas-Martín, López-Bueno [16] found a lower minimum mortality temperature among women, indicating greater vulnerability. However, they also found that Spanish women had adapted more to heat in the period 1983–2018 [16]. A review of sex/gender disparities in mortality rates found that the majority of analyses indicated a higher mortality rate among women, also suggesting greater vulnerability to extreme heat [10]. A recent report by a German health insurance company found that 33% of women had experienced heat-related health problems, compared to 15% of men [40]. Due to biological differences, women may be more vulnerable to heat due to lower heat evaporation (sweating), greater adipose tissue and lower muscle mass as well as decreased peripheral blood perfusion [16]. These disparities may be even greater in postmenopausal women, as estrogen and progesterone play an important role in regulating body temperature [10, 41].

The present mediation analysis indicates that exposure to heat at home is a pathway through which social inequalities are connected to the health consequences of climate change. In urban contexts, deprived neighborhoods have been found to experience a more pronounced increase in heat compared to wealthier neighborhoods, as these areas have higher settlement densities and fewer green spaces [7]. Furthermore, social deprivation has been linked to lower housing quality [7] and more crowded living spaces [42]. In a nationwide German study, low-income households exhibited reduced adaptive capacity to heat [24]. Outdoor and indoor temperatures were found to be weakly associated but to differ significantly depending on building characteristics [43]. As elderly persons spend most of their time at home [19], increased indoor temperature may be a more reliable predictor of health consequences or mortality than outdoor temperature [43]. It is also noteworthy that the majority of studies in this field focus on urban health [22]. Nonetheless, previous work indicated more adverse health outcomes in urban contexts [22, 23] and the results of our sensitivity analysis suggest a similar pattern, as the effect from domestic heat exposure can be considered slightly more pronounced within urban residents.

### Strengths and limitations

DigiHero is a large, population-based cohort study in which cross-sectional and longitudinal data on the health of the German population is regularly collected online. Unlike most scientific research, our focus was on domestic heat exposure, since outdoor heat exposure may limit the ability to assess heat-related health risks, particularly among older adults. While the model fit indices indicated an excellent fit across all three analyzed models, we have to acknowledge as one limitation that our sample was somewhat selected, as our education variable, in contrast to the standard categorization, classifies ISCED 1 to 4 as “low”. Since data were collected via an online survey, limited digital literacy may have prevented some people from participating, particularly older adults. Additionally, participation in the survey may reflect individual awareness of about heat exposure and may have induced a selection bias. As further limitation, self-reported exposure and outcomes may be biased by health and heat awareness. Furthermore, we did not collect data on the amount of time participants spent indoors, and therefore cannot (a) evaluate a dose-response relationship, or (b) assess whether observed sex/gender differences may be attributable to women spending more time indoors.

Although the participants were highly educated and wealthy (the majority reported an equivalized net income higher than the German median [44]), social inequalities were found to be present regarding heat exposure/effects. Sex/gender bias in the self-reported exposure and outcome may have led to an overestimation of differences between men and women, but this was only of a qualitative nature, as the stratified analysis compared men and women within their respective sex/gender groups. Recall bias may have been limited as the survey was conducted in August, meaning not much time had passed since the most recent heat experience.

## Conclusions

Our study revealed inequalities in the health consequences of heat exposure among older adults in Germany. Domestic heat exposures contribute to these inequalities, as indicated by mediation effects among women. Findings among men were inconsistent, likely reflecting differences in awareness and health response to heat. These results are consistent with existing evidence on social inequalities in heat exposure and contribute to the previous body of evidence showing that outdoor temperature is associated with adverse health consequences and mortality. Our findings highlight the importance of ensuring equitable access to cool spaces, particularly indoors, for older adults during hot days or heat waves. Political decision-making should support structural-level cooling (e.g., [45]) and reduce inequalities in housing quality. Further research should aim to objectively (and simultaneously) measure and distinguish between indoor and outdoor temperature and examine sex/gender differences in heat-related health consequences in more detail.

## Supplementary Information

Below is the link to the electronic supplementary material.

Supplementary Material 1

## Data Availability

De-identified individual data and analytic code are available upon reasonable request from the corresponding author after publication, subject to approval by the DigiHero Use and Access Committee and a data-use agreement.

## Abbreviations

RMSEA: Root mean square error of approximation
ISCED: International Standard Classification of Education
SEM: Structural Equation Model
CFI: Comparative Fit Index
RIT: Ratio of the indirect and total effect
