# Supplementary material for: Heat exhaustion, domestic heat exposure and socioeconomic disparities among older adults: a cross-sectional mediation analysis from Germany
Source: Int J Equity Health. 2026 Jul 7;25:165. doi: 10.1186/s12939-026-02934-8 (PMC13339498; doi:10.1186/s12939-026-02934-8)
Supplement: Supplementary file 1 — Supplementary Material 1 [file 12939_2026_2934_MOESM1_ESM.docx]

**Additional Files**

**Table A1: Questionnaire in German and English**

| **No** | **Item (German)** | **Answer option (German)** | **Item (English)** | **Answer option (English)** | **Source** |
| --- | --- | --- | --- | --- | --- |
| 1 | Bitte geben Sie Ihr Geschlecht an | 1 = männlich; 2 = weiblich; 3 = divers; 4 = keine Angabe | Please specify your gender | 1 = male; 2 = female; 3 = diverse; 4 = prefer not to say; | German National Cohort (NAKO)^1^ |
| 2 | Wann sind Sie geboren? | Datum | What is your date of birth? | date | German National Cohort (NAKO)^1^ |
| 3 | Sind Sie zurzeit erwerbstätig? | 1 = ja, Voll- oder in Teilzeit erwerbstätig; 2 = nein (einschließlich: SchülerInnen oder Studierende, die nicht gegen Geld arbeiten, VorruheständlerInnen, RentnerInnen ohne Nebenverdienst) | Are you currently employed? | 1 = Yes, employed full-time or part-time 2 = No (including students or persons not in paid employment, early retirees, and retirees without additional income) | German National Cohort (NAKO)^1^ |
| 4 | Welche beruflichen Ausbildungsabschlüsse haben Sie? | 1 = Noch in beruflicher Ausbildung (Berufsvorbereitungsjahr, Ausbildung, Praktikum, Studium); 2 = SchülerIn und besuche eine berufsorientierte Aufbau-, Fachschule o.ä.; 3 = Keinen beruflichen Abschluss und bin nicht in beruflicher Ausbildung; 4 = Beruflich-betriebliche Berufsausbildung (Lehre) abgeschlossen; 5 = Beruflich-schulische Ausbildung abgeschlossen (Berufsfachschule, Handelsschule, Vorbereitungsdienst für den mittleren Dienst in der öffentlichen Verwaltung); 6 = Ausbildung an einer Fachschule der DDR abgeschlossen; 7 = Ausbildung an einer Fach-, Meister-,Technikerschule, Berufs- oder Fachakademie abgeschlossen; 8 = Bachelor an einer (Fach-) Hochschule abgeschlossen; 9 = Fachhochschulabschluss (z.B. Diplom, Master); 10 = Universitätsabschluss (z.B. Diplom, Magister, Staatsexamen, Master); 11 = Promotion; 12 = Einen anderen beruflichen Abschluss; 13 = Weiß nicht; 14 = Keine Angabe; 15 = welchen anderen beruflichen Abschluss haben Sie? | What is your highest level of education or vocational training? | 1 = Still in vocational training (pre-vocational year, apprenticeship, internship, or university/college studies); 2 = Student attending a vocational-oriented secondary or technical school; 3 = No vocational qualification and not currently in vocational training; 4 = Completed vocational apprenticeship (company-based); 5 = Completed vocational school-based training (vocational school, commercial school, preparatory service for mid-level public administration); 6 = Completed training at a DDR-era technical school; 7 = Completed training at a technical, master, or professional/fach academy; 8 = Bachelor’s degree from a university or university of applied sciences; 9 = Degree from a University of Applied Sciences (e.g., Diploma, Master’s degree); 10 = University degree (e.g., Diploma, Magister, State Examination, Master’s degree); 11 = Doctorate (PhD); 12 = Another vocational or professional qualification; 13 = Don’t know; 14 = Prefer not to say; 15 = What other vocational or professional qualification do you have? | German National Cohort (NAKO)^1^ |
| 5 | Wie hoch ist das durchschnittliche monatliche Nettoeinkommen Ihres  Haushalts insgesamt?  (Unter durchschnittlichem monatlichem Nettoeinkommen Ihres  Haushalts ist die Summe zu verstehen, die sich aus Lohn, Gehalt,  Einkommen aus selbstständiger Tätigkeit (durchschnittliche  Nettobezüge, das heißt abzüglich der Betriebsausgaben und der  Steuern), Rente oder Pension ergibt. Rechnen Sie bitte auch die  Einkünfte aus öffentlichen Beihilfen, Einkommen aus Vermietung  und Verpachtung, Vermögen, Wohngeld, Kindergeld und sonstige  Einkünfte hinzu und ziehen Sie dann Steuern und  Sozialversicherungsbeiträge ab.) | 1 = unter 1 250 Euro 1 250; 2 = bis unter 1 750 Euro;  3 = 1 750 bis unter 2 250 Euro;  4 = 2 250 bis unter 3 000 Euro;  5 = 3 000 bis unter 4 000 Euro;  6 = 4 000 bis unter 5 000 Euro;  7 = 5 000 Euro und mehr  8 = Ich möchte nicht antworten. | What is the total average monthly net income of your household?  (By the total average monthly net income of your household, we mean the sum of wages and salaries, income from self-employment (average net earnings, i.e., after deducting business expenses and taxes), and pensions or retirement benefits. Please also include income from public assistance, rental income, assets, housing benefits, child benefits, and other sources, and then subtract taxes and social security contributions.) | 1 = Less than €1250 2 = €1250 to less than €1750 3 = €1750 to less than €2250 4 = €2250 to less than €3000 5 = €3000 to less than €4000 6 = €4000 to less than €5000 7 = €5000 or more 8 = Prefer not to say | German National Cohort (NAKO)^1^ |
| 6 | Wenn es ein richtig heißer Sommer ist, hat Ihr Haus / Ihre Wohnung in den Bereichen, in denen Sie sich meistens aufhalten, angenehme Temperaturen? | 1 = ja; 2 = nein; 3 = teils/teils; | If it is a really hot summer, does your house/apartment have comfortable temperatures in the areas where you spend most of your time? | 1 = Yes 2 = No 3 = Partly | Self-generated |
| 7 | Welche Temperaturen haben Sie in Ihrem normalen Schlafraum, wenn es ein richtig heißer Sommer ist? | 1 = unter 20 Grad; 2 = 20-24 Grad; 3 = 25-29 Grad; 4 = 30-34 Grad; 5 = über 35 Grad; | What temperatures do you have in your usual bedroom when it is a really hot summer? | 1 = Below 20 °C 2 = 20–24 °C 3 = 25–29 °C 4 = 30–34 °C 5 = Above 35 °C | Self-generated |
| 8 | Können Sie dann in einen Raum zum Schlafen ausweichen, wo es weniger heiß ist? | 1 = ja; 2 = nein; | Can you move to another room for sleeping where it is less hot? | 1 = yes; 2 = no | Self-generated |
| 9 | Ich habe in diesem Sommer unter folgenden gesundheitlichen Beeinträchtigungen gelitten: | Jedes dieser Symptome war auf einer Skala von 0 = nie bis 5 = sehr oft einzuordnen  Abgeschlagenheit und Müdigkeit; Schlechtere Konzentrationsfähigkeit; Schlechte Schlafqualität; Kreislaufbeschwerden und Schwindel; Kopfschmerzen; Unruhegefühl; Geschwollene Füße oder Beine; Kurzatmigkeit; Vermehrte Allergie (wenn Sie keine Allergie haben, kreuzen Sie "nie" an) | During this summer, I have suffered from the following health problems: | Each of these symptoms should be rated on a scale from 0 = never to 5 = very often:  Fatigue and tiredness; Reduced ability to concentrate; Poor sleep quality; Circulatory problems and dizziness; Headaches; Restlessness; Swollen feet or legs; Shortness of breath; Increased allergy symptoms (if you do not have allergies, select “never”) | Adapted from Buhtz et al. 2024^2^ |
| 10 | Waren die von Ihnen genannten gesundheitlichen Beeinträchtigungen hitzebedingt?  (Filterfrage; wurde nicht erfragt, wenn Frage Nummer 9 mit „nie“ beantwortet wurde) | Waren die folgenden Symptome hitzebedingt? Pro Symptom: 1 = ja; 2 = nein  Abgeschlagenheit und Müdigkeit; Schlechtere Konzentrationsfähigkeit; Schlechte Schlafqualität; Kreislaufbeschwerden und Schwindel; Kopfschmerzen; Unruhegefühl; Geschwollene Füße oder Beine; Kurzatmigkeit; Vermehrte Allergie | Were the health problems you mentioned caused by heat? | Were the following symptoms caused by heat? (Per symptom: 1 = Yes; 2 = No)  Fatigue and tiredness; Reduced ability to concentrate; Poor sleep quality; Circulatory problems and dizziness; Headaches; Restlessness; Swollen feet or legs; Shortness of breath; Increased allergy symptoms | Adapted from Buhtz et al. 2024^2^ |

^1^ Dragano N, Reuter M, Greiser KH, Becher H, Zeeb H, Mikolajczyk R, et al. Soziodemografische und erwerbsbezogene Merkmale in der NAKO Gesundheitsstudie. Bundesgesundheitsblatt - Gesundheitsforschung - Gesundheitsschutz. 2020;63(3):267-78.

^2^ Buhtz C, Frese T, Jahn P, Kantelhardt E, Kuhlmann A, Lückmann SL, Meyer G, Meyer-Feil T, Schildmann J, Steckelberg A, Knöchelmann A (2024). Subjektive gesundheitliche Beeinträchtigung und assoziierende Faktoren in der Hitzeperiode des Sommers 2022 – Ein Online-Survey, Gesundheitswesen. DOI: 10.1055/a-2332-0059

| **Table A2: Sample characteristics for sensitivity analysis** | | | | |
| --- | --- | --- | --- | --- |
|  | **Rural** | **Urban** | **Total** | |
| **N** | 684 (47.0%) | 771 (53.0%) | 1455 (100.0%) | |
| **Age** |  |  |  | |
| Mean (SD) | 72.7 (5.1) | 72.9 (5.0) | 72.8 (5.0) | |
| **Sex/Gender** |  |  |  | |
| Men | 328 (48.0%) | 338 (43.8%) | 666 (45.8%) | |
| Women | 356 (52.0%) | 433 (56.2%) | 789 (54.2%) | |
| **Education according to ISCED** |  |  |  | |
| Low (ISCED 1-4) | 195 (30.1%) | 213 (29.3%) | 408 (29.7%) | |
| Medium (ISCED 5-6) | 143 (22.1%) | 132 (18.2%) | 275 (20.0%) | |
| High (ISCED 7-8) | 309 (47.8%) | 382 (52.5%) | 691 (50.3%) | |
| **Equivalized net household income [€]** |  |  |  | |
| Less than 2,000 | 283 (44.4%) | 246 (35.1%) | 529 (39.5%) | |
| 2,000-3,000 | 203 (31.8%) | 218 (31.1%) | 421 (31.5%) | |
| 3,000-4,000 | 100 (15.7%) | 137 (19.6%) | 237 (17.7%) | |
| 4,000 and more | 52 (8.2%) | 99 (14.1%) | 151 (11.3%) | |
| **Domestic Heat Exposure^1^** |  |  |  | |
| 0 | 477 (69.8%) | 521 (67.7%) | 998 (68.7%) | |
| 1 | 183 (26.8%) | 192 (24.9%) | 375 (25.8%) | |
| 2-3 | 23 (3.4%) | 57 (7.4%) | 80 (5.5%) | |
| **Frequency of Heat Exhaustion^2^** |  |  |  | |
| Mean (SD) | 1.5 (2.1) | 1.6 (2.3) | 1.6 (2.2) | |
| ^1^ Sum of exposures to elevated temperatures during the night, limited access to cooler environments during the night, and presence of uncomfortable indoor temperatures at home during the day.  ^2^ Included symptoms were dizziness, headache, and shortness of breath.  SD = Standard Deviation; ISCED = International Standard Classification of Education | | | |  |

| **Table A3: Results from urban/rural-stratified sensitivity analysis** | | | |
| --- | --- | --- | --- |
| **Path** | **All Participants**  **(n= 1455)** | **Urban (n= 771)** | **Rural (n= 684)** |
| **Direct effects** | β (95% CI) | β (95% CI) | β (95% CI) |
| *on domestic heat exposure* |  |  |  |
| Education | -0.02 (-0.07, 0.04) | -0.01 (-0.09, 0.07) | -0.02 (-0.10, 0.06) |
| Income | -0.10 (-0.15, -0.05) | -0.12 (-0.18, -0.04) | -0.09 (-0.17, -0.02) |
|  |  |  |  |
| *on heat exhaustion* |  |  |  |
| Education | -0.08 (-0.13, -0.02) | -0.11 (-0.19, -0.04) | -0.04 (-0.12, 0.04) |
| Income | -0.08 (-0.13, -0.02) | -0.08 (-0.15, -0.01) | -0.07 (-0.15, -0.001) |
| Domestic Heat | 0.09 (0.04, 0.15) | 0.12 (0.05, 0.19) | 0.04 (-0.03, 0.12) |
| Age | 0.004 (-0.05, 0.06) | -0.003 (-0.09, 0.08) | 0.004 (-0.06, 0.09) |
|  |  |  |  |
| *on income* |  |  |  |
| Education | 0.28 (0.22, 0.32) | 0.29 (0.23, 0.36) | 0.25 (0.18, 0.33) |
| Age | 0.02 (-0.04, 0.07) | 0.01 (-0.06, 0.08) | 0.03 (-0.05, 0.11) |
|  |  |  |  |
| *on education* |  |  |  |
| Age | 0.06 (0.01, 0.11) | 0.01 (-0.05, 0.08) | 0.11 (0.03, 0.18) |
|  |  |  |  |
| **Total effect on heat exhaustion** | β (95% CI) | β (95% CI) | β (95% CI) |
| Income | -0.09 (-0.14, -0.03) | -0.10 (-0.17, -0.02) | -0.08 (-0.15, -0.004) |
| Education | -0.10 (-0.16, -0.05) | -0.14 (-0.21, -0.07) | -0.06 (-0.14, 0.02) |
|  |  |  |  |
| **Indirect effect on heat exhaustion (via domestic heat exposure)** | β (95% CI) | β (95% CI) | β (95% CI) |
| Income | -0.01 (-0.01, -0.003) | -0.01 (-0.03, -0.004) | -0.004 (-0.02, 0.002) |
| Education | -0.001 (-0.01, 0.003) | -0.001 (-0.01, 0.01) | -0.001 (-0.01, 0.002) |

| **Table A4: Results from complete cases sensitivity analysis** | | | |
| --- | --- | --- | --- |
| **Path** | **All Participants**  **(n= 1242)** | **Men (n= 581)** | **Women (n= 661)** |
| **Direct effects** | β (95% CI) | β (95% CI) | β (95% CI) |
| *on domestic heat exposure* |  |  |  |
| Education | -0.01 (-0.08, 0.04) | 0.03 (-0.05, 0.12) | -0.04 (-0.11, 0.05) |
| Income | -0.09 (-0.14, -0.03) | -0.09 (-0.16, 0.004) | -0.08 (-0.15, -0.01) |
|  |  |  |  |
| *on heat exhaustion* |  |  |  |
| Education | -0.08 (-0.14, -0.02) | -0.02 (-0.10, 0.07) | -0.09 (-0.16, -0.01) |
| Income | -0.08 (-0.13, -0.02) | -0.07 (-0.15, 0.01) | -0.07 (-0.14, -0.001) |
| Domestic Heat | 0.09 (0.04, 0.16) | -0.04 (-0.13, 0.04) | 0.17 (0.09, 0.24) |
| Age | 0.01 (-0.04, 0.07) | 0.07 (-0.01, 0.16) | -0.01 (-0.09, 0.08) |
|  |  |  |  |
| *on income* |  |  |  |
| Education | 0.28 (0.22, 0.33) | 0.26 (0.17, 0.33) | 0.28 (0.20, 0.35) |
| Age | 0.01 (-0.06, 0.06) | -0.02 (-0.10, 0.06) | 0.03 (-0.05, 0.10) |
|  |  |  |  |
| *on education* |  |  |  |
| Age | 0.06 (0.01, 0.12) | 0.08 (0.004, 0.16) | 0.02 (-0.06, 0.10) |
|  |  |  |  |
| **Total effect on heat exhaustion** | β (95% CI) | β (95% CI) | β (95% CI) |
| Income | -0.09 (-0.14, -0.03) | -0.07 (-0.15, -0.02) | -0.09 (-0.16, -0.01) |
| Education | -0.11 (-0.16, -0.05) | -0.04 (-0.12, 0.05) | -0.11 (-0.19, -0.04) |
|  |  |  |  |
| **Indirect effect on heat exhaustion (via domestic heat exposure)** | β (95% CI) | β (95% CI) | β (95% CI) |
| Income | -0.01 (-0.02, -0.002) | 0.004 (-0.002, 0.02) | -0.01 (-0.03, -0.002) |
| Education | -0.001 (-0.01, 0.003) | -0.002 (-0.01, 0.001) | -0.01 (-0.02, 0.01) |
